# Efficacy and safety of Infliximab in systemic sarcoidosis according to GenPhenReSa organ-involvement phenotype: a retrospective study of 55 patients

**DOI:** 10.1186/s12931-024-02758-6

**Published:** 2024-03-14

**Authors:** Etienne Rivière, Wendy Jourde, Noémie Gensous, Xavier Demant, Emmanuel Ribeiro, Pierre Duffau, Patrick Mercié, Jean-François Viallard, Estibaliz Lazaro

**Affiliations:** 1 Internal Medicine and Infectious Diseases unit, Haut-Leveque Hospital, University Hospital Centre of Bordeaux, F33604 Pessac Cedex, France; 2 INSERM U1034, Bordeaux University, F33604 Pessac Cedex, France; 3 Department of Internal Medicine and Clinical Immunology, Saint Andre Hospital, University Hospital Centre of Bordeaux, F33000 Bordeaux, France; 4 ImmunoconcEpT; FHU ACRONIM, UMR CNRS 5164, Bordeaux University, F33000 Bordeaux, France; 5 Respiratory Diseases unit, Haut-Leveque Hospital, University Hospital Centre of Bordeaux, CIC 1401, F33604 Pessac Cedex, France; 6 Univ. Bordeaux, INSERM, BRIC, U1312, F33000 Bordeaux, France

**Keywords:** Sarcoidosis, Infliximab, Disease clusters, Infection

## Abstract

**Background:**

Infliximab is currently recommended as a third-line treatment for refractory sarcoidosis. Data in function of clinical phenotype are currently lacking. We evaluated patients’ characteristics and responses to infliximab according to their GenPhenReSa cluster.

**Methods:**

We evaluated clinical and biological characteristics of patients diagnosed with sarcoidosis who received infliximab between September 2008 and April 2019 at our centre.

**Results:**

Fifty-five patients (median disease duration, 87 months) received infliximab: 48 (87%) as a second- or third-line treatment, and 7 (13%) as a first-line treatment. After a median duration of 12 months, 24 (45%) and 14 (25%) patients achieved complete and partial responses, respectively, together with a significant decrease in the number of affected organs and tapering of steroid doses. All patients with neurosarcoidosis (OR 17), 90% in group 2 (ocular-cardiac-cutaneous-CNS, OR 7.4), and approximately two-thirds of those in groups 1 (abdominal organs), 4 (pulmonary-lympho-nodal), and 5 (extrapulmonary), achieved a response, whereas patients in group 3 (musculoskeletal-cutaneous) had a treatment-failure OR of 9. Infliximab could be stopped after complete remission was achieved in 7 patients: 4 relapsed after a median of 6 months. Overall, 36% of patients experienced serious adverse events, mainly infections, which led to treatment cessation in 29% of patients and caused two deaths.

**Conclusions:**

Other than patients with musculoskeletal-cutaneous involvement (group 3), infliximab led to a good response for patients with CNS (group 2) and liver (group 1) organ-predominant sarcoidosis. However, it led to serious infections and merely suspended sarcoidosis, so further research on factors predictive of relapse is needed.

## Key message

Infliximab is highly effective in patients with CNS (group 1) and liver (group 2) organ-predominant sarcoidosis.

Infliximab is poorly effective in patients with musculoskeletal-cutaneous involvement (group 3).

Infectious episodes are frequent and can be severe in this immunocompromised population.

## Introduction

Sarcoidosis is a systemic granulomatous disease that can potentially lead to serious complications when affecting crucial organs, such as the brain, heart, lungs, kidneys, liver, or eyes. It is a great mimicker [1] in medicine, and complications can occur if diagnosis is delayed; physicians should therefore be aware of any clinical sign suggestive of sarcoidosis [2]. The disease can affect any organ but typically involves the lungs and has a highly heterogeneous clinical presentation. To enhance analysis of disease phenotypes and responses to treatment, the GenPhenReSa project in 2018 [3] suggested classifying patients into five clusters according to predominant organ involvement: abdominal organs (kidneys/liver/spleen [AO]), ocular–cardiac–cutaneous–central nervous system (eye/heart/skin/salivary glands/CNS [OCCC]), musculoskeletal–cutaneous (MSC), pulmonary-lympho-nodal (PLN), and extra-pulmonary (EP).

The cause of sarcoidosis is unknown but its pathophysiology is being better understood. Pivotal roles are played by genetic background; macrophages; and T helper, including T helper 17 (Th17), and T-regulator (Treg) cells in granuloma formation in response to one or more environmental antigens [4]. The function of TNF-α in the genesis of a non-caseating epithelioid granuloma is now established; [5–7] it stimulates the recruitment of mononuclear cells via chemokine secretion, the emergence of giant cells, and allows cell cohesion inside the granuloma [8]. In the last 10 years, the anti-TNF-α therapies have caused clinical improvement in some patients with sarcoidosis [8–10]. Infliximab is currently recommended as a third-line treatment, notably when an advanced phenotype is evidenced after failing to control the acute and chronic phases of sarcoidosis [11]. Data are lacking regarding the characteristics of patients who respond to infliximab, its efficacy against lesions in rarely involved organs, treatment duration, and interruption modalities.

We conducted a retrospective study to analyse the infliximab response, tolerance, and treatment modalities in adult patients with systemic sarcoidosis according to the five GenPhenReSa clusters.

## Methods

### Patients

We retrospectively collected patients’ data from the medical records of our hospital between September 2008 and April 2019. All patients gave consent to participate. Patients who received infliximab for sarcoidosis during that period were identified in the files of the central pharmacy. Sarcoidosis was diagnosed according to the WASOG criteria [13] as a clinical phenotype suggestive of sarcoidosis, confirmed by a biopsy evidencing non-caseating epithelioid granulomas, with any differential diagnosis excluded. Patients who received infliximab for < 3 months were excluded from the efficacy analysis. For each patient, organ lesions were identified according to the WASOG criteria, and the phenotypic presentation was categorised according to the five GenPhenReSa clusters. Severe organ damage was defined according to the level of clinical or radiological anatomical or functional impairment, or a severe degree of inflammation or tissue destruction/fibrosis on biopsy. Immunosuppressive treatments received concomitantly with infliximab were recorded, as was any change in the steroid dose when such treatments were added or stopped. Patients receiving > 10 mg steroids daily before infliximab was introduced were considered steroid dependent.

### Primary and secondary endpoints

The primary criteria were the efficacy and safety of infliximab at the end of follow-up, in patients who received infliximab for ≥ 3 months according to the five clusters of clinical presentation. The end of follow-up was defined as “treatment interruption,” whatever the justification, or the need for treatment upgrade, or the date of censorship (April 2019). Treatment upgrade was the addition or increase of an immunosuppressive agent or steroids in association with infliximab. Response was defined as described previously [8]: complete response, disappearance of clinical signs (excluding sequelae) upon daily use of < 10 mg steroids; partial response, improvement of clinical and para-clinical parameters upon > 50% reduction of the initial steroid dosage; and non-response, when patients did not fulfil the complete or partial response criteria.

Secondary criteria were assessment of the efficacy for each organ involved at the end of follow-up (using the criteria mentioned above), efficacy and safety in patients who received infliximab as a first-line treatment, and the number of relapses after treatment interruption. Adverse events (AEs) were retrospectively collected at each follow-up appointment, and graded from 1 to 5 according to the Common Toxicity Criteria for Adverse Events (CTCAE, http://ctep.cancer.gov/protocolDevelopment/electronic_applications/ctc.htm).

### Ethics

The study was conducted in accordance with the Declaration of Helsinki and according to the French Legislation MR-004 (https://www.legifrance.gouv.fr/jorf/id/JORFTEXT000037187498) and was approved by the institutional review board of our hospital.

### Statistical analysis

Quantitative variables were expressed in mean (min/max) for age, and in median with interquartile range (IQR) for all other variables. The Fisher’s exact test, chi-square, Mann–Whitney, and Kruskal–Wallis tests for non-parametric variables were performed using GraphPad Prism v. 6. A *p*-value < 0.05 defined significance.

## Results

### Patients’ characteristics

This study included 55 patients, the baseline characteristics of whom are listed in Table 1. The median age at diagnosis was 40 years, and 47 years at the time of infliximab treatment. Only 3 patients were ancient (2) or active smokers (1). The median disease duration before starting infliximab was 45 months (IQR 14–108). When infliximab was started, patients had a median of 1 organs involved (IQR 1–2, stage-1 lung involvement was counted as lymph nodes involvement). Forty-six patients (84%) had severe disease defined as severe damage to one organ, or at least three organs involved. According to the GenPhenReSa clustering, 9 patients were in group 1 (AO), 21 in group 2 (OCCC), 10 in group 3 (MSC), 9 in group 4 (PLN), and 6 in group 5 (EP).

**Table 1:** Baseline characteristics of the 55 patients at infliximab initiation

Variable	N (%) [IQR]
**Age at start of infliximab, mean (years, min/max)**	47 (18–80)
**Sex (men/women)**	27/28 (49/51)
**Ethnicity**	
Caucasian Maghrebis West-Indies	39 (71) 8 (14.5) 8 (14.5)
**Median disease duration (median, IQR)**	45 [14–108]
**Median number of involved organs (IQR)**	1 [1–2]
**Main involved organs**	
Lungs (stages II to IV) Central nervous system Lymph nodes Joints Bones Liver Eye(s) Skin Muscles Spleen Hypercalcemia Kidney(s) Peripheral nervous system Gut Heart Ear-nose-throat	16 (29) 14 (25.5) 10 (18) 8 (14.5) 8 (14.5) 8 (14.5) 6 (11) 5 (9) 5 (9) 5 (9) 3 (5.5) 3 (5.5) 3 (5.5) 2 (3.5) 2 (3.5) 1 (2)
**Median number of previous treatment lines (IQR)**	2 [1–3]
**Previous immunosuppressive treatments**	
Steroids Methotrexate Cyclophosphamide Mycophenolate mofetil Azathioprine Hydroxychloroquin Others	47 (85) 23 (42) 11 (20) 10 (18) 6 (11) 6 (11) 4 (7)

### Treatments received

The main causes of treatment switch to infliximab, which were sometimes present in combination in the same patient, were previous treatment failure for 35 patients (63.6%) with a median number of previous treatments of 2 (steroids for 98% and at least one immunosuppressant for 75%), steroid dependence (dose ≥ 10 mg/day) in 24 (44%), sarcoidosis recurrence in 11 (20%), and AEs in 11 patients (20%). Of note, 25% of patients only received steroids with or without infliximab at first line and no further immunosuppressive agent because of a contra-indication. The organ involvement that triggered infliximab use was the central nervous system (24%), multi-systemic disease (18%), lung (15%), eye (9%), and other (liver, muscles, joints, peripheral nervous system). Eight and three patients concomitantly received methotrexate and mycophenolate mofetil, respectively, including two who newly started this treatment (Table 1).

Seven patients received infliximab as a first-line therapy. Their mean age was 45 years, and four were of Afro-Caribbean ancestry. Four patients had biopsy-proven severe liver damage with major cholestasis, moderate hepatic cytolysis, and no sign of acute liver-failure, and a median of two organs involved (including lymph nodes, spleen, bone, and/or lungs). Two patients had isolated neurosarcoidosis with brain and medullar lesions, and one had extensive lymph nodes and bone infiltration. They received a median steroid dose of 30 mg/day (IQR-20-60), a higher proportion than the other patients (*p* = 0.01), with more patients requiring increased doses of steroids at infliximab initiation (*p* = 0.04), and three receiving pulses of methylprednisolone. None had previously received an immunosuppressive drug.

Infliximab was administered at 5 mg/kg on average every 4 to 6 weeks (median, 5 weeks). When infliximab was introduced, 26 patients (47%) had an increase in steroid dose, and 16 (29%) received pulses of methylprednisolone. The median steroid dose was 20 mg/day (IQR 10–40) when infliximab was started (Table 2).

**Table 2:** Indications for infliximab use and concomitant treatment(s)

Variable	N (%)
Indications for infliximab (may be combined)	
Previous treatments failure	35 (64)
Steroid dependence (≥ 10 mg/day)	24 (44)
Adverse events related to previous treatments	11 (20)
Recurrence	11 (20)
Involved organs that triggered the use of infliximab	
Central nervous system Multi-systemic Lungs Eye Liver Joints Muscles Peripheral nervous system Other (heart, ear-nose-throat, gut)	16 (29) 10 (18) 8 (15) 5 (9) 3 (6) 3 (6) 2 (5) 2 (5) 4 (7)
Concomitant treatments received	
Infliximab *Mean dose (mg/kg)* *Median interval between two infusions (weeks, IQR)* Oral steroids *Median dose (mg/day, IQR)* *Patients who required increased dose at initiation* Pulses of methylprednisolone Immunosuppressant Methotrexate Mycophenolate mofetil	55 (100) 5 5 [4–6] 47 (85) 20 [10–40] 26 (47) 16 (29) 11 (20) 8 (14.5) 3 (5)

### Overall efficacy

**Fig. 1:**
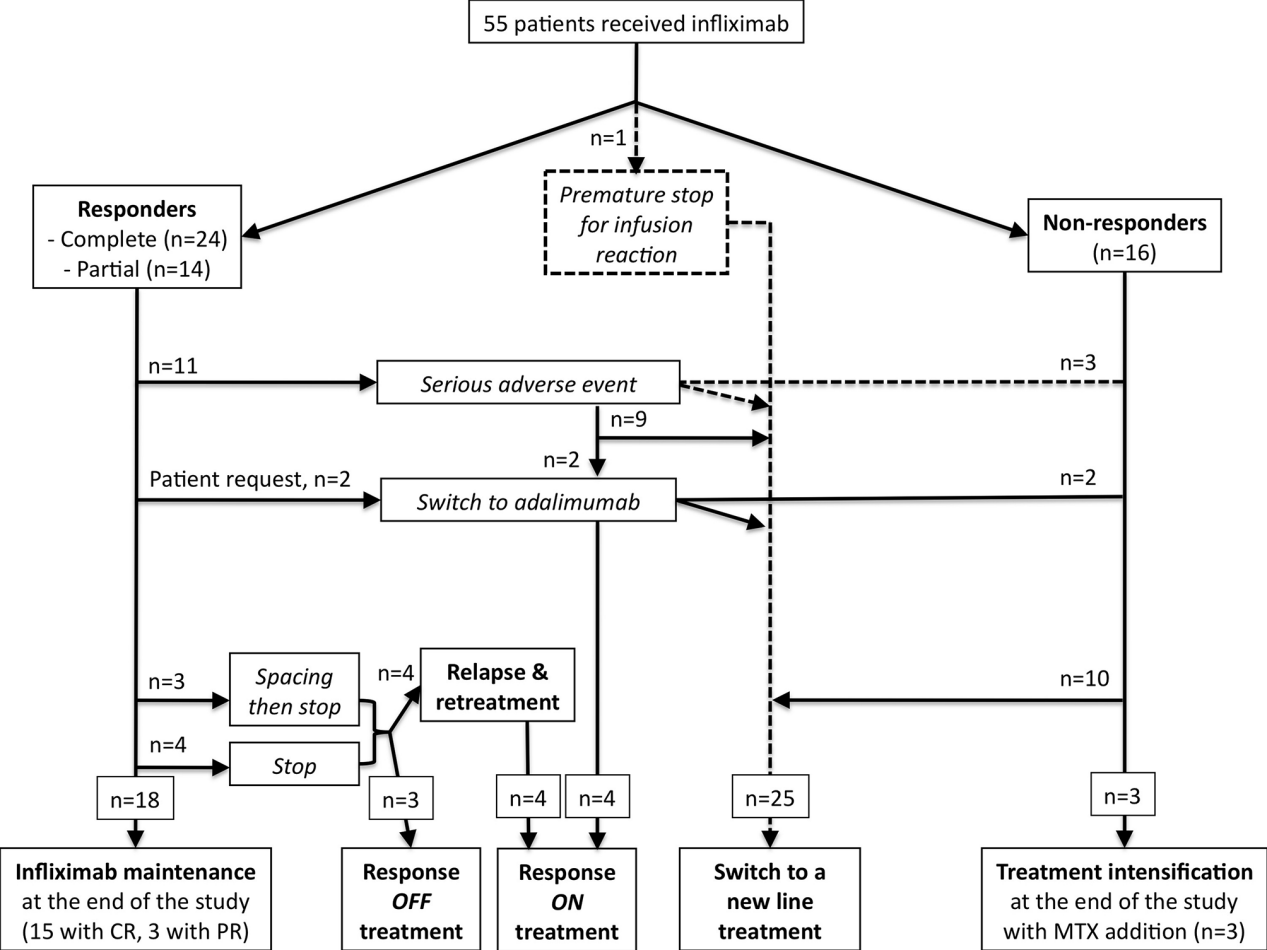
Study flow chart. *MTX*, *methotrexate; CR*, *complete response; PR*, *partial response*

Overall infliximab efficacy was assessed in 54 of 55 patients, because 1 patient experienced an infusion-related AE after three infusions that led to treatment interruption. After a median follow-up of 12 months (IQR: 6–20 months), 24 patients (44%) achieved a complete response and 14 (26%) achieved a partial response, for an overall efficacy of 70%. By contrast, 16 patients did not respond to infliximab (Fig. 1). Table 3 lists the patients’ characteristics according to response to infliximab. Responders had a significantly smaller number of damaged organs (0.4 vs. 1.8, *ρ* < 0.0001), and a decreased steroid dose (5.3 vs. 30 mg/day at initiation, *ρ* < 0.0001). This difference was also significant in patients who received steroids for an acute flare that triggered infliximab (6.3 vs. 13 mg/day, *ρ* = 0.01). Concomitant immunosuppressants were stopped in two patients.

**Table 3:** Patients’ characteristics according to response to infliximab, among the 54 patients eligible for efficacy assessment. *CRP*, *C-reactive protein. Chi-square or Fisher’s exact and Mann-Whitney tests*

Characteristics	Responders (*N* = 38)	Non-responders (*N* = 16)	ρ-value
**Age, median (years, min-max)**	47,5 [18–79]	44 [28–80]	1
**Sex (men/women)**	20/18 (53/47)	6/10 (37.5/62.5)	0.4
**Ethnicity**			
Caucasian Maghrebis West-Indies	26 (69) 7 (18) 5 (13)	12 (75) 1 (6) 3 (19)	0.7 0.4 0.7
**Median disease duration (months, IQR)**	45 [13.5–98]	38,5 [17–100]	0.8
**Main involved organ**			
Central nervous system Lungs Systemic disease Liver Eye Peripheral nervous system Muscles Joints Others	13 (34) 4 (10.5) 4 (10.5) 4 (10.5) 3 (8) 3 (8) 2 (5) 1 (3) 4 (10.5)	0 3 (19) 6 (37.5) 0 2 (12.5) 0 1 (6) 3 (19) 1 (6)	**0.006** 0.4 0.06 0.6 0.3 0.07 1 0.5 0.8
**Lymphopenia** CD4^+^ count (×10^9^/L)	25 (69) 0.54	10 (67) 0.72	1 0.3
**Increased gamma globulin levels**	5 (18.5)	4 (36)	0.4
**Inflammation with elevated CRP**	19 (54)	6 (37.5)	0.4
**Elevated angiotensin-converting enzyme**	13 (37)	5 (36)	1
**Median number of previous therapies (IQR)**	2 [1–2.75]	2 [1–2.25]	0.7
**Number of patients who required an increased doses of oral steroids**	20 (53)	5 (31)	0.2
**Median dose of oral steroids (mg/day, IQR)**	17.5 [2.5–40]	20 [12–51]	0.3
**Pulses of methylprednisolone**	13 (34)	2 (12.5)	0.2
**Concomitant immunosuppressant**	8 (21)	3 (19)	1

During follow-up in the responders group, 15 (27%) and 3 (5.5%) patients maintained complete and partial responses, respectively, with a median steroid dose of 5 mg/day. Infliximab was stopped in 20 responders: 7 who had stable disease (4 complete and 3 partial responses) after a median treatment duration of 23 months, 11 for AEs after a median of 11.5 months (IQR 6–16), and in 2 patients who requested adalimumab to avoid hospital stay who maintained response. In the 18 other patients: 2 patients who had sever AEs received adalimumab and maintained response, 3 patients maintained sustained response off-treatment, and 13 experienced relapse after a median of 4.5 months. In detail, the seven responders were receiving steroids at &amp;lt; 5 mg/day and two also received methotrexate and mycophenolate mofetil, respectively; these two patients did not relapse. Three patients underwent progressive spacing between infusions before infliximab interruption. Four patients relapsed after a median of 7.5 months, all with the same initial organ involvement, and all achieved complete responses after infliximab retreatment. The other three patients were still in remission after a median follow-up of 2.2 years. Sarcoidosis involved the eye, kidneys, and CNS in these three patients, and all were in complete response when infliximab was stopped. At the end of follow-up, 25 patients (46%) were still receiving infliximab, including 4 retreated patients and 3 non-responders who needed additional methotrexate to control the disease.

The 16 patients (29%) who did not respond to infliximab had a median number of involved organs of 2 (IQR 1–2) at the end of follow-up, with a median steroid dose of 8.5 mg (44% received &amp;lt; 5 mg/day). Systemic disease, as well as joint and muscle damage, was predominant in this group. Infliximab was stopped for inefficacy after a median of 6 months (IQR 6-13.75). Three patients received cyclophosphamide, which led to complete responses in a patient with ear-nose-throat (ENT) involvement and another with kidney and muscle damage; the third patient did not tolerate treatment, which was prematurely stopped. Two patients received adalimumab but did not respond. Methotrexate was introduced in the three other patients still receiving infliximab, leading to a complete response in a patient with extensive skin disease and another with severe eye involvement, but no response in the third patient with skin lesions. Six (86%) of seven patients responded to infliximab as a first-line therapy.

### Efficacy according to involved organ(s) in the GenPhenReSa phenotypes

All patients with CNS involvement responded to infliximab (OR, 17; 95% CI, 0.97–314; *ρ* = 0.006), with positive (PPV) and negative (NPV) predictive values of 100% and 39%, respectively. Eight patients had liver involvement (two evidenced via ^18^FDG-PET and six proven by biopsy), with a complete response in seven.

Because the many effects of TNF-α might lead to different clinical phenotypes with corresponding pathophysiologies, [3] we evaluated the response to infliximab according to the five GenPhenReSa clusters (Table 4). There were significantly more Afro-Caribbean patients in group 1 (*ρ* = 0.01) and a trend towards more women in groups 3 and 5. More patients in group 5 had lymphopenia < 1 × 10^9^/L (*ρ* = 0.03). Disease duration before infliximab initiation tended to be shorter and inflammation was greater in patients in groups 1 and 5. Steroids were increased in patients in groups 1 and 2 (*ρ* = 0.04), and the mean dose tended to be lower in these groups when infliximab was started. 90% of patients in group 2 (OCCC) responded to infliximab (OR = 7.4; 95% CI: 1.5–37.2, *ρ* = 0.01) with 14 (67%) complete responders, 77% in group 1 (AO) with 4 (44%) complete responders, and approximately two-thirds of those in groups 4, and 5, with 2 (22%) and 4 (67%) of compete responders respectively. In addition, 70% of patients in group 3 (MSC) did not respond to infliximab (OR = 9; 95% CI: [1.9–42.2], *ρ* = 0.004, Table 5 ).

**Table 4:** Patients’ clinical and biological characteristics according to GenPhenReSa group at infliximab initiation. *CRP*, *C-reactive protein. Non-parametric Kruskal-Wallis test*

Characteristics	Group 1 (AO, *N* = 9)	Group 2 (OCCC, *N* = 21)	Group 3 (MSC, *N* = 10)	Group 4 (PLN, *N* = 9)	Group 5 (EP, *N* = 6)	ρ-value
**Age, mean (years, min-max)**	49.2 [32–79]	49.1 [13–80]	47.9 [28–70]	51.3 [39–78]	49.3 [40–69]	0.9
**Sex (male/female)**	4/5	11/10	3/7	7/2	2/4	0.27
**Ethnicity**						
Caucasian Maghrebis West-Indies	3 (33) 1 (12) 5 (55)	17 (81) 3 (14) 1 (5)	9 (90) 0 (0) 1 (10)	5 (55) 3 (33) 1 (12)	5 (83) 1 (17) 0 (0)	**0.01**
**Median disease duration (months, IQR)**	36 [7.5–74]	65 [14–188]	49.5 [21.25–180]	85 [30.5–121]	15.5 [3.5–40]	0.09
**Lymphopenia** CD4^+^count (×10^9^/L)	7 (77) 1.3	11 (52) 1.37	4 (40) 1.48	8 (89) 1.13	6 (100) 0.8	**0.03** 0.14
**Increased gamma globulin levels**	3 (33)	1 (5)	2 (20)	2 (22)	1 (17)	0.34
**Inflammation with elevated CRP**	7 (77)	8 (38)	3 (33)	4 (44)	4 (67)	0.18
**Elevated angiotensin-converting enzyme**	3 (33)	6 (28)	2 (20)	5 (55)	2 (33)	0.55
**Median number of previous therapies (min-max)**	1 [0–2]	2 [1–2.5]	2 [1–4]	2 [2–3]	2 [0.75–3.25]	0.22
**Number of patients who required an increased dose of steroids**	6 (67)	14 (67)	2 (20)	2 (22)	2 (33)	**0.04**
**Median dose of oral steroids (mg/day, IQR)**	0 [0–8.75]	9 [0–17.5]	7.5 [0–16.25]	15 [0–20]	30 [3.75–51.25]	0.18
**Pulses of methylprednisolone**	2 (33)	9 (43)	1 (10)	1 (12)	3 (50)	0.16
**Concomitant immunosuppressant**	0 (0)	4 (19)	4 (40)	1 (12)	2 (33)	0.21

**Table 5:** Response to infliximab according to GenPhenReSa sarcoidosis cluster among the 54 patients eligible for efficacy assessment. *Fisher’s exact test*

Cluster	Response	Failure	ρ-value
**Group 1 “AO,”** *n* **= 9** *Abdominal Organs (kidney-liver-spleen)*	7 (77%)	2 (23%)	0.7
**Group 2 “OCCC,”** *n* **= 21** *Ocular-Cardiac-Cutaneous-CNS*	19 (90%)	2 (10%)	**0.01**
**Group 3 “MSC,”** *n* **= 10** *Musculoskeletal-Cutaneous*	3 (30%)	7 (70%)	**0.004**
**Group 4, “PLN,”** *n* **= 8** *Pulmonary-Lympho-Nodal*	5 (62.5%)	3 (37,5%)	0.7
**Group 5 “EP,”** *n* **= 6** *Extra-Pulmonary*	4 (67%)	2 (33%)	1

*CNS*, *central nervous system*

### Factors predictive of response

Taking into account the small number of patients, the duration of infliximab treatment and progressive spacing of infusions did not prevent relapse. We did not find biological markers predictive of relapse or treatment cessation. A CRP level &amp;gt; 0.8 mg/mL predicted response to infliximab in 138 patients,[14] but the median CRP levels in this study were 5.5 mg/mL (IQR 1.4–12) and 3.4 mg/mL (IQR 2.1–9.9) in responders and non-responders, respectively, with a minimum CRP level of 0.9 mg/mL in non-responders. A maximum standardised uptake value (SUV_max_) of six or more mediastinal lymph nodes was suggested to predict relapse. Among the 25 patients (45%) who underwent ^18^fluorodeoxyglucose positron emission tomography (^18^FDG-PET) before infliximab was started, 18 and 7 patients were responders and non-responders, respectively, of whom 9 responders and 2 non-responders had an SUV_max_ of < 6 (*ρ* = 1). [15]

### Safety

**Fig. 2:**
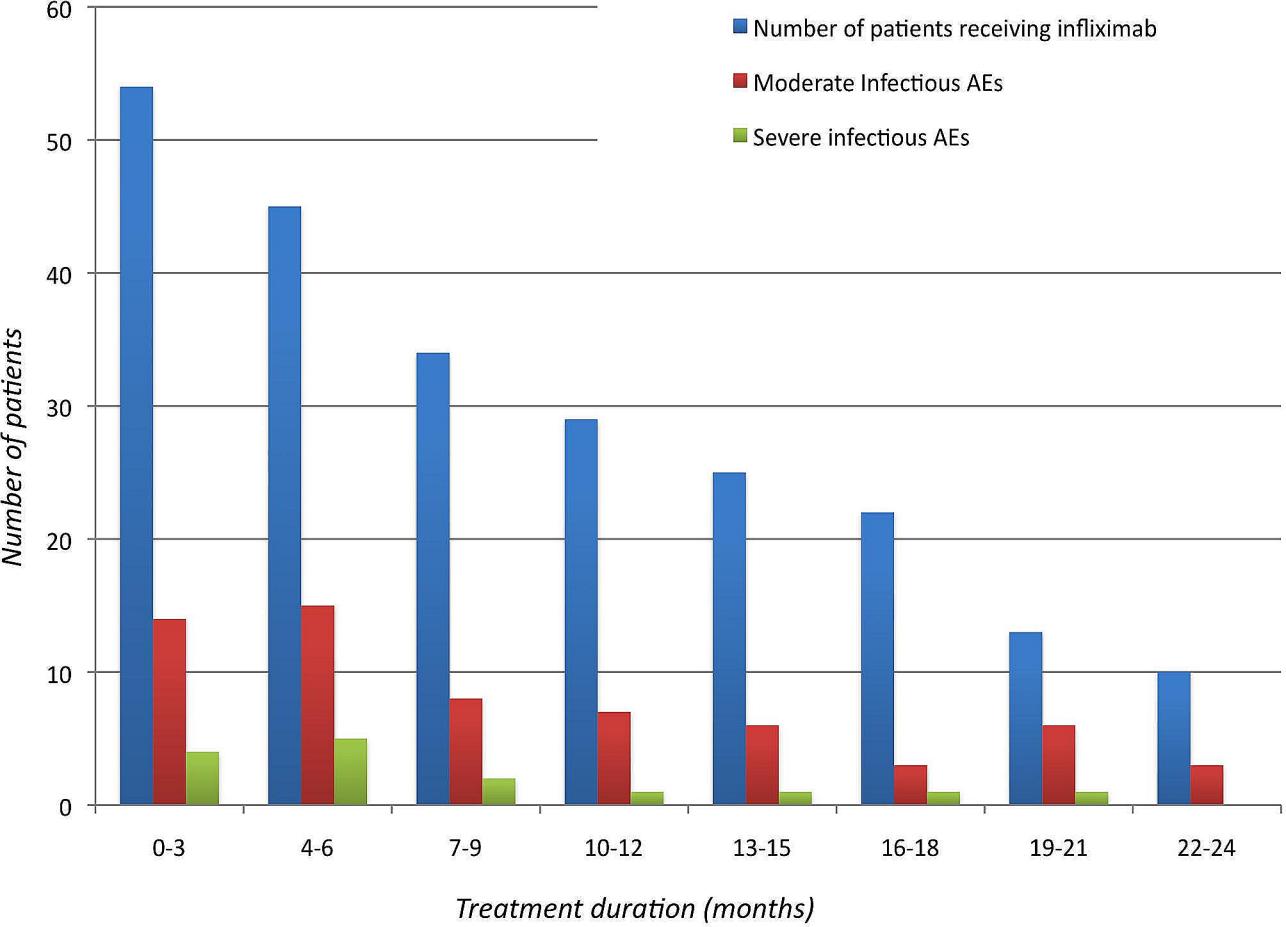
Infectious AEs in patients receiving infliximab

During follow-up, 42 patients (76%) experienced 85 AEs (AEs) leading to treatment interruption in 16 (29%) and death in 2 (4%) (linked to severe pneumonia). We noted 73 infections, mainly bacterial infections of the lungs or ENT, which led to hospitalisation in 13 patients (24%) because of a severe infection of the lungs (4), gut (2), urinary tract (2), bones (2), skin (1), or diffuse infection due to *Mycobacterium bovis* (1) unrelated to BCG instillation, and *Listeria* sp. (1). We noted viral infections in seven patients (13%) due to cytomegalovirus (but no CMV disease), respiratory syncytial virus, human papilloma virus, and herpes zoster virus. We detected vaginal yeast infections in four women (7%). Infectious AEs tended to be more numerous during the first 6 months, when the steroid dose was highest (Fig. 2). We found no link between age, disease duration, lymphocyte count, number of organs involved, number of previous treatment lines, steroid dose, and associated immunosuppressant when infliximab was started between patients who did and did not experience at least one infectious event. The two deceased patients were a 64-year-old man with joint and muscle involvement, and a 70-year-old woman with isolated pulmonary sarcoidosis. Both died of severe pneumonia after 4 months of treatment and had previously received steroids and methotrexate (the latter stopped once infliximab was started), with a steroid dose of 25 mg in the first and 10 mg/day in the second patient at the time of the infectious event.

There were no significant differences in terms of infectious AEs according to GenPhenReSa phenotype due to the small number of subjects in each group. Patients who experienced severe infectious AEs tended to be older (59 vs. 45 years, *ρ* = 0.09), but there were no differences in terms of disease duration, steroid dose, pulses of methylprednisolone, or concomitant immunosuppressive treatment.

Six patients developed infliximab intolerance: three infusion-related reactions, two severe hepatic cytolyses, and one severe digestive intolerance. Two patients experienced alloimmunisation against infliximab after 18 months of treatment, including one patient who was concomitantly receiving methotrexate. All of these eight patients experienced treatment interruption. The seven patients who received infliximab as a first-line treatment had AEs, including three severe infections that did not lead to treatment interruption, and one thyroid cancer.

## Discussion

We analysed a cohort of 55 patients with a median disease duration of 87 months who received infliximab as a first-line treatment or for refractory or steroid-dependent sarcoidosis. Infliximab was overall effective for 70% of the cases, allowing significant steroid sparing. All patients with neurosarcoidosis (OR, 17), 90% of those in group 2 (OCCC cluster; OR, 7.4), and approximately two-thirds of patients in groups 1 (AO), 4 (PLN), and 5 (EP) achieved a response, whereas patients in group 3 (MSC) had an OR for failure of 9. When infliximab was stopped in seven responding patients, four relapsed, with no identifiable predictive factor, no preventive effect of spacing infusions, and full recovery of infliximab efficacy once restarted. Finally, the treatment morbidity rate was high.

The overall efficacy of infliximab in refractory systemic sarcoidosis varies from 58 to 100% [12–15, 8]. We found a higher rate of complete response (44%) than previously reported, [8] probably because of the smaller number of patients with refractory sarcoidosis and the smaller number of involved organs in this study.

Regarding organ involvement, several studies have reported ambiguous results for the lungs [16, 17] and skin, [18] but interesting results for CNS, [19, 20] heart, [21, 22] or eye [23] involvement, as in this work. Notably, 73% of patients with a PLN presentation achieved a response, as previously suggested [24] but contradicting the findings of two controlled trials [16, 17]. However, efficacy was assessed after 6 weeks only in the first trial, [16] and the second included only patients with stabilised sarcoidosis [17]. Yet, it has been suggested that severe active lung involvement with inflammation is predictive of response to anti-TNF-α therapies, [24, 25] which might explain the higher rate of response in this work.

Infliximab was less efficacious for treating joint, muscle, or skin lesions of the patients in group 3. However, data on infliximab and joint involvement in sarcoidosis are lacking, [26] because chronic sarcoid arthritis [27] and dactylitis [28] are rare and because many patients might have benefited from therapies that also acted on joints (steroids, methotrexate). Data are also scarce regarding muscle involvement; one study found no change in the ePOST [8] and in another, only 18% of patients responded [12]. An insufficient response of skin lesions to infliximab is surprising and can be explained by premature stoppage of infliximab at 6 months by physicians in this study; a longer duration is required. In a study in which only 46% of patients responded at 6 months, 79% responded at 12 months [18]. Moreover, our patients had mild to moderate lesions, yet infliximab has greater efficacy for severe lesions such as lupus pernio [29]. 

In our cohort of eight patients with liver involvement, infliximab had a remarkable and promising effect. One study reported 47% efficacy for small liver lesions, which did not trigger infliximab use [12]. Other reported cases have confirmed this [30, 31]. 

Concomitant immunosuppressants could have increased the response rate because four patients initiated this therapy simultaneously with infliximab. However, in our work, these factors were not predictive of response, similar to Jamilloux et al. [8] and were added in only 20% of patients, compared to 50–75% of patients in other studies, [13, 17, 19] suggesting that these patients had better responses to infliximab. It has been proposed that steroids at > 20 mg/day reduce TNF-α expression and could decrease infliximab efficacy in terms of respiratory tests [32]. However, these patients might have had severe disease. By contrast, more responders received steroids in this study, suggesting that steroids do not affect the response to infliximab.

Our findings confirm that infliximab merely suspended sarcoidosis: 67% of responders who stopped it relapsed after a median of 4.5 months. We did not identify factors predictive of relapse, as reported by others [19, 24]. In Jamilloux et al., the relapse rate was low (14%) after a median of 14 months, possibly because many patients were still receiving steroids and/or immunosuppressants [8]. Predominant CNS involvement was predictive of a response to infliximab (OR, 17), and predominant musculoskeletal cutaneous involvement was predictive of failure (OR, 9) in univariate analyses. The ongoing TAWIS study might provide insight into how and when to stop infliximab (NCT05689879).

Six patients received another TNF antagonist in this study, with the same response (either positive or negative) that infliximab as previously suggested by two studies [8, 33]. In responders, it seems possible to switch infliximab for another anti-TNF monoclonal antibody (except etanercept which has a lower efficacy) [9, 10] to improve comfort and decrease cost, however the evidence for this strategy is very low.

One third of the patients experienced severe AEs that led to hospitalisation and/or treatment interruption, and death in two patients was linked to infection. Patients who experienced these AEs tended to be nonsignificantly older than those who did not develop serious AEs. In addition to greater immunosuppressive use in patients with sarcoidosis, these results might be linked to more severe disease-induced immunosuppression in sarcoidosis than in other autoimmune diseases. Indeed, a meta-analysis of 453 patients who received infliximab for rheumatoid arthritis, Crohn’s disease, and ulcerative colitis followed for up to 3 years, showed that only 21% of patients had infections, of which 3.4% were severe [34]. Because we and others [18] observed no association between the advent of severe infectious episodes and the initial steroid dose, concomitant immunosuppressants, or number of previous treatment lines, infliximab itself could be responsible for these events. Indeed, the seven patients who received infliximab as a first-line treatment also experienced serious infections. By contrast, Baughman et al. [17] found no difference in terms of infectious AEs between infliximab and placebo groups, possibly because patients received infliximab every 6 weeks.

Interestingly, seven patients received infliximab as a first-line treatment; six showed a favourable response but five experienced serious infectious AEs. Given its efficacy in severe extrapulmonary presentations such as the CNS or liver, with possible sequelae, infliximab can be a first-line therapy, balanced with the infectious risk. Predictive factors, such as the levels of dendritic cells [39] and monocytes/macrophages in blood or bronchoalveolar lavage,[40] could enable the tailoring of treatment.

This study had several limitations. The assessment of infliximab efficacy for each involved organ in accordance with the GenPhenReSa cluster classification was performed on a small number of patients in each group. As a retrospective analysis of medical records, this study was subject to evaluation bias. However, the monocentric approach enabled collection of homogeneous data. A major issue was the lack of objective scores or criteria for evaluating sarcoidosis severity. The ePOST score has been used in several studies to assess extrapulmonary lesions but is subjective because it requires global assessment of the disease by a physician. Its use in retrospective studies is problematic, so we employed a global evaluation approach, as used by Jamilloux et al., that combined clinical and paraclinical evaluation, together with steroid sparing.

This study also had several strengths. We evaluated infliximab use in real life in the second-largest cohort published to date, which included patients with various disease phenotypes [8, 15, 35]. In addition, this was the first study to associate real-life results with GenPhenReSa clusters. Other published cluster phenotypes have shown more or less convergent results [36, 37]. These clusters were initially published to assist clinical trials in sarcoidosis by creating homogeneous groups of patients with various clinical phenotypes. Moreover, a recent meta-analysis proposed investigation of the effect of anti-TNF-α agents in patients with pulmonary involvement separately from those with other conditions, [10] which we suggest for patients with the MSC phenotype.

## Conclusion

Infliximab shows promise for treating patients with sarcoidosis, particularly with CNS and liver involvement, but not musculoskeletal or cutaneous involvement. However, it can cause serious infections and only suspends sarcoidosis; therefore, predictive factors for relapse should be the focus of further research. Moreover, trials on infliximab as a first-line treatment in patients with severe disease and a favourable benefit-risk balance (probably younger patients) are needed.

## Data Availability

All data can be available upon request to the corresponding author.
